# Recombinant expression of an l‐amino acid oxidase from the fungus *Hebeloma cylindrosporum* in *Pichia pastoris* including fermentation

**DOI:** 10.1002/mbo3.1112

**Published:** 2020-08-27

**Authors:** Marc Christian Heß, Svenja Bloess, Joe Max Risse, Karl Friehs, Gabriele Fischer von Mollard

**Affiliations:** ^1^ Biochemistry III Department of Chemistry Bielefeld University Bielefeld Germany; ^2^ Fermentation Engineering Faculty of Technology Bielefeld University Bielefeld Germany

## Abstract

l‐amino acid oxidases (LAAOs) are flavoenzymes that catalyze the oxidative deamination of l‐amino acids to the corresponding α‐keto acids, ammonia, and hydrogen peroxide. Here, we show the overexpression, purification, and the characterization of LAAO4 from the fungus *Hebeloma cylindrosporum* in the yeast *Pichia pastoris* with a 9His‐tag and compare this with the recently characterized 6His‐*hc*LAAO4 expressed in *E*.* coli*. The expression of the enzyme with an ER‐signal sequence in *P*.* pastoris* resulted in a glycosylated, secreted protein. The enzymatic activity without activation was higher after expression in *P*.* pastoris* compared to *E*.* coli*. Due to treatment with acidic pH, a striking increase of activity could be detected for both expression systems resulting in similar specific activities after acid activation. Regarding the substrate spectrum, temperature stability, *K*_m,_ and *v*_max_ values, *hc*LAAO4 showed very few differences when produced in these two expression systems. A higher yield of *hc*LAAO4 could be obtained by fermentation.

## Graphical abstract

*hc*LAAO4 was successfully expressed in *Pichia pastoris* as a secreted, glycosylated enzyme. *K* *_m_* and *v* *_max_* values, substrate spectrum, and temperature stability were similar when expressed in *E*.* coli* or in *P*.* pastoris*. Expression of *hc*LAAO4 could be drastically increased when fermented in bioreactors compared to shaking flasks in both expression systems with much higher values obtained regarding activity per gram dry mass and activity per liter for *P*.* pastoris*.

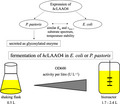

## 1 INTRODUCTION

l‐amino acid oxidases (LAAOs, EC 1.4.3.2) are oxidoreductases, which catalyze the oxidative deamination of l‐amino acids to imino acids. Due to spontaneous hydrolysis, the corresponding α‐keto acid and ammonia are formed. As a byproduct, hydrogen peroxide is formed during regeneration of the non‐covalently bound cofactor flavin adenine dinucleotide (FAD; Pollegioni, Motta, & Molla, 2013). LAAOs are found in several organisms like mammals, bacteria, algae, and fungi even though the functions differ in different organisms. Snake venom LAAOs (SV‐LAAO) are the best‐characterized enzymes and can cause apoptosis, edema, or hemolysis (Ali et al., 2000; Du & Clemetson, 2002; Suhr & Kim, 1996; Weia et al., 2007). LAAOs possess antimicrobial or antiparasitic functions in fish, molluscs, fungi, and bacteria due to H_2_O_2_ formation (Kasai et al., 2010; Tong, Chen, Shi, Qi, & Dong, 2008; Yang et al., 2005, 2011). Furthermore, the formation of ammonia is an important nitrogen source for anabolic reactions in fungi (Nuutinen & Timonen, 2008).

For industry, LAAOs are of great interest because of the formation of α‐keto acids or the possibility to obtain enantiomerically pure d‐amino acids from racemic mixtures by enzymatic resolution. Unfortunately, recombinant expression of LAAOs with broad substrate spectrum has been difficult to obtain for a biotechnological application (Hossain et al., 2014). Using different bacterial sequences, an ancestral LAAO has been designed and expressed efficiently in *E*.* coli*, which has a broad substrate specificity but low thermal stability (Nakano, Minamino, Hasebe, & Ito, 2019). Only d‐amino acid oxidases (DAAOs) can be produced in a sufficient amount and quality for a biotechnological application (Pollegioni & Molla, 2011).

Recently, we were able to express two fungal LAAOs in *E*.* coli*, a gram‐negative bacterium. The LAAO1 from the fungus *Rhizoctonia solani* (*rs*LAAO1) was expressed as a fusion protein with maltose‐binding protein (Hahn, Neumeister, et al., 2017) and the LAAO4 from the fungus *Hebeloma cylindrosporum* (*hc*LAAO4) with an N‐terminal 6His‐tag (Bloess et al., 2019). *rs*LAAO1 showed low activity and has to be activated by low amounts of SDS (Hahn, Hertle, et al., 2017). Because of the possible interference of SDS in process development, we identified another LAAO with a broader substrate spectrum. *hc*LAAO4 can be activated by brief exposure to acid pH and stays in the active conformation (Bloess et al., 2019). Here, we show the expression of *hc*LAAO4 in *Pichia pastoris* as secreted protein and describe differences of the enzyme expressed in *E*.* coli* and in *P*.* pastoris* with regard to glycosylation, acid activation, substrate spectrum, kinetic parameters, and temperature stability. Moreover, we use fermentation in bioreactors to produce a higher cell density and achieving a higher yield of recombinantly expressed enzyme.

## 2 MATERIALS AND METHODS

### 2.1 Amplification and cloning of 9His‐*hc*LAAO4

The synthetic gene of the *Hebeloma cylindrosporum* LAAO4 (*hc*LAAO4) without the putative ER signal sequence (GenBank accession number MH751433, Bloess et al., 2019) was amplified with a forward primer (AAGAATTC **CATCATCATCACCATCACCACCACCAT**ATGGCTGACACTACTTCCGTTC) to introduce an *Eco*RI recognition site (underlined sequence) followed by a 9His‐tag (bold) and a codon for a methionine residue upstream of the *hc*LAAO4 coding sequence. The reverse Primer (AAGGCGGCCGC **TTA**AACGGAAAC) contained the stop‐Codon (bold) and the *Not*I site (underlined sequence). The PCR product was cloned via an intermediate step into pGEM‐TEasy (Promega), digested with *EcoR*I and *Not*I, and ligated into the *EcoR*I and *Not*I digested pPIC9K (MPI), which encodes the prepro‐sequence of α‐factor, yielding pSBL11. The four N‐glycosylation sites predicted by NetNGlyc were changed into alanine residues one after the other via overlap PCR with the primers listed in Table A1 resulting in pMCH24.

### 2.2 Genome integration and selection of clones

For genome integration, pSBL11 or pMCH24 were linearized at the *Sal*I site and transformed into the spheroplasts of *P*.* pastoris* SMD1163 yeast strain (Life Technologies) for integration into *HIS4* locus. After electroporation, 1 M ice‐cold sorbitol was added immediately and the cells were spread on MD‐His agar plates (0.17% yeast nitrogen base, 0.5% biotin, 2% glucose, amino acids, and 1.5% Agar) and were incubated at 30°C for 2 days, according to Wu & Letchworth 2004. The clones were spread onto YEPD agar plates with increasing concentration of G‐418 (Geneticin, Calbiochem) (1, 2.5, 5, 7.5, and 10 mg/ml) and cultured at 30°C for several days. The selection was done according to Scorer, Clare, McCombie, Romanos, & Sreekrishna, 1994. Clones were screened for the highest 9His‐*hc*LAAO4 expression after induction with methanol.

### 2.3 Expression of 9His‐*hc*LAAO4 in shaking flasks

Clones were cultured in 20 ml BMGY medium (2% peptone, 1% yeast extract, 100 mM potassium phosphate pH 6.0, 1.34% yeast nitrogen base, 0.4 µg/ml biotin, and 1% glycerol) at 30°C for at least 16 hr. Cells were centrifuged and diluted into 500 ml BMMY medium (2% peptone, 1% yeast extract, 100 mM potassium phosphate pH 6.0, 1.34% yeast nitrogen base, 0.4 µg/ml biotin, and 1% methanol) for 72 hr at 15°C. Methanol was added every 24 hr to a final concentration (v/v) of 1% to keep the methanol concentration up. Cells were pelleted (4°C, 4600 *g*, 10 min), and the supernatant consisting of the medium with secreted 9His‐*hc*LAAO4 was stored at 4°C and used for purification of the enzyme. Cells were spheroplasted and 0.5 ml ruptured with a Precellys24 bead homogenizer with four ceramic beads (1.4 mm) by two repeats of twice 20 s at 6500 rpm interrupted by a 10‐second break to analyze for 9His‐*hc*LAAO4 remaining within the cells. A soluble fraction was separated from larger particles by centrifugation (4°C, 18,000 *g*, 5 min).

### 2.4 Fermentation of *P*.* pastoris* in bioreactors

Cells were incubated at 30°C in 300 ml BMGY medium (2% peptone, 1% yeast extract, 100 mM potassium phosphate pH 6.0, 1.34% yeast nitrogen base, 0.4 µg/ml biotin, and 1% glycerol) overnight. 9‐*hc*LAAO4 was expressed in a 7 L‐NLF (Bioengineering AG) bioreactor with an initial volume of 3 L BSM‐medium (26.7 ml/L *o*‐phosphoric acid (86%), 0.93 g/L CaSO_4_, 18.2 g/L K_2_SO_4_, 14.9 g/L MgSO_4_·7H_2_O, 4.13 g/L KOH, 40 g/L glycerol (99%), 4.35 ml/L PTM trace salts (6 g/L CuSO_4_, 0.08 g/L NaI, 3 g/L MnSO_4_·H_2_O, 0.2 g/L MoO_3_·H_2_O, 0.02 g/L H_3_BO_3_, 0.5 g/L CoCl_2_, 20 g/L ZnCl_2_, 65 g/L FeSO_4_·7H_2_O, 0.2 g/L biotin, 5 ml/L sulfuric acid)). The pH was kept at 6.0 by automated addition of 25% (w/w) ammonia solution and 10% (w/w) phosphoric acid, respectively. To avoid foaming Pluronic^®^, PE 8100 was added automatically. Fermentations were performed at 30°C with 0.2 bar overpressure and an aeration rate of 5 NL/min. The initial stirrer frequency was set to 200 min^−1^ and speed was increased in steps of 2% when the relative dissolved oxygen saturation (rDOS) fell below 30% (batch‐phase). A first supplementary feeding started automatically after 5 hr when rDOS raised from 30% to 60% for the first time and stopped each time when rDOS felt below 60% again. When 1 L of 500 g/L glycerol (99%) with 1.2% (v/v) PTM trace salts was added during the first feeding phase the cultivation temperature was lowered to 15°C and a second feeding phase with a solution of 1 L methanol with 1.2% (v/v) PTM trace salts was started after 47 hr of cultivation. A methanol concentration of 0.3% (v/v) in the bioreactor was maintained by feed control via a methanol sensor (Alcosens; Heinrich Frings GmbH & Co. KG). The expression was performed for about 4–5 days. In total, 735 g (928 ml) of methanol solution was added.

### 2.5 Fermentation of *E*.* coli* in bioreactors

Expression of 6‐*hc*LAAO4 in *E*.* coli* Arctic Express (DE3) cells (Ferrer, Chernikova, Timmis, & Golyshin, 2004) in shaking flask was recently described in Bloess et al., 2019. For fermentation in 3.7 L KLF (Bioengineering AG) bioreactors, a culture of transformed Arctic Express (DE3) cells in 40 ml LB medium with 50 μg/ml kanamycin and 25 μg/ml gentamycin was injected into a bioreactor filled with 2 L HSG medium (13.5 g/L soy peptone, 7 g/L yeast extract, 14.9 g/L glycerol (99%), 2.5 g/L NaCl, 2.3 g/L K_2_HPO_4_, 1.5 g/L KH_2_PO_4_, and 0.14 g/L MgSO_4_·H_2_O) containing 50 μg/ml kanamycin and 25 μg/ml gentamycin. The pH was kept at 7.0 by automated addition of 2 M sodium hydroxide solution (25% (w/w) ammonia solution during fed‐batch procedures) and 10% (w/w) phosphoric acid, respectively. To avoid foaming Pluronic^®^, PE 8100 was added automatically. Fermentations were performed with 0.2 bar overpressure and an aeration rate of 2 NL/min. The initial stirrer frequency was set to 200 min^−1^ and speed was increased in steps of 2% when rDOS felt below 30%. In the case of fed‐batch procedures, feeding of a solution of 600 g/L glycerol, 90 g/L yeast extract, and 1 g/L MgSO_4_·H_2_O was started when rDOS raised from 30% to 60% for the first time and stopped each time when rDOS felt below 60% again. Several growth periods were tested ranging from 28 to 70 hr at 30°C or 37°C. Expression was induced with 0.2 mM IPTG after cooling down the fermentation broth to 11°C for 17–25 hr. After fermentation, cells were disrupted by the French press and the enzyme purified according to Bloess et al., 2019.

### 2.6 Purification of the secreted 9‐*hc*LAAO4

Purification of the 9‐*hc*LAAO4 fusion protein was carried out at 4°C. After the removal of the cells by centrifugation, the supernatant with the secreted 9‐*hc*LAAO4 in the medium was loaded onto Ni^2+^‐NTA resin. The flow‐through was caught, mixed, and reloaded onto Ni^2+^‐NTA resin via a peristaltic pump for 24 hr and a flow velocity of 1 ml/min. The column was washed with 40 column volumes (cv) of ‐washing buffer (50 mM Na_2_HPO_4_ pH 7.0, 300 mM NaCl, and 20 mM imidazole). After washing, 9‐*hc*LAAO4 was eluted with His buffer (50 mM Na_2_HPO_4_ pH 7.0, 300 mM NaCl) containing different concentrations of imidazole (50, 100, 250, and 500 mM). The enzyme was concentrated via ultrafiltration (Vivaspin 6 30.000 MWCO, Sartorius), rebuffered into HEPES buffer pH 7.0 (100 mM HEPES, 150 mM NaCl) and stored at 4°C. Size exclusion chromatography was performed according to Bloess et al., 2019 with an Ettan LC (GE Healthcare Life Sciences, Chicago, IL, USA) on a Superdex^®^ 200 *Increase* 10/300 GL column (GE Healthcare Life Sciences, Chicago, IL, USA).

### 2.7 Enzymatic assay

Determination of the enzymatic activity of 9‐*hc*LAAO4 or 6‐*hc*LAAO4 by measuring the initial rate of H_2_O_2_ production was done by coupled peroxidase/*o*‐dianisidine assay with 10 mM l‐amino acid as described (Hahn, Neumeister, et al., 2017). The standard assay mixture contained 10 mM l‐glutamine, 50 mM TEA/HCl buffer (pH 7.0), 0.2 mg/ml of *o*‐dianisidine, 5 U/ml peroxidase and *hc*LAAO4 in limiting amounts (0.75 µg in a 200 µl assay). Reactions were carried out in 96‐well plates at 30°C in a Tecan Spark microplate reader at 436 nm. One unit was defined as the amount of enzyme that catalyzes the conversion of 1 μmol l‐amino acid per minute. Initial velocities of H_2_O_2_ production were determined with 16 different l‐amino acid concentrations between 0.02 and 20 mM for the untreated and acid‐activated 9‐*hc*LAAO4. *K*_m_ and *v*_max_ values were calculated from Hanes–Woolf plots. To determine the pH optimum, the enzyme was treated with buffers at different pH‐values (Sörensen glycine‐HCl buffers pH 2–2.5 (Sörensen, 1909), 200 mM Na_2_HPO_4_, 100 mM citric acid buffers pH 3–6.5 (McIlvaine, 1921), 50 mM TEA buffers pH 7–9 and 50 mM glycine pH 9.5–11) before the assay. Activation was tested by preincubation for 10 min at the indicated pH and activity determined at pH 7.0. For determining the temperature stability, the enzymes were incubated at the respective temperature in the TEA/HCl buffer for 1 hr.

### 2.8 Deglycosylation of 9‐*hc*LAAO4

Deglycosylation was performed by PNGaseF (NEB). The PNGaseF mixture contained 100 mM EDTA pH 8.0, 100 mM Tris‐HCl pH 8.0, 1 µM PMSF in DMSO, 1% (w/v) CHAPS, and 500 U PNGaseF. The digestion was done overnight at 37°C. As control samples, PNGaseF was replaced with water and these samples were also incubated at 37°C or 4°C overnight. Samples were analyzed by sodium dodecyl sulfate‐polyacrylamide gel electrophoresis (SDS‐PAGE, Laemmli, 1970) and stained with Coomassie Brilliant Blue R‐250.

## 3 RESULTS

### 3.1 Expression and purification of 9‐*hc*LAAO4

**FIGURE 1:**
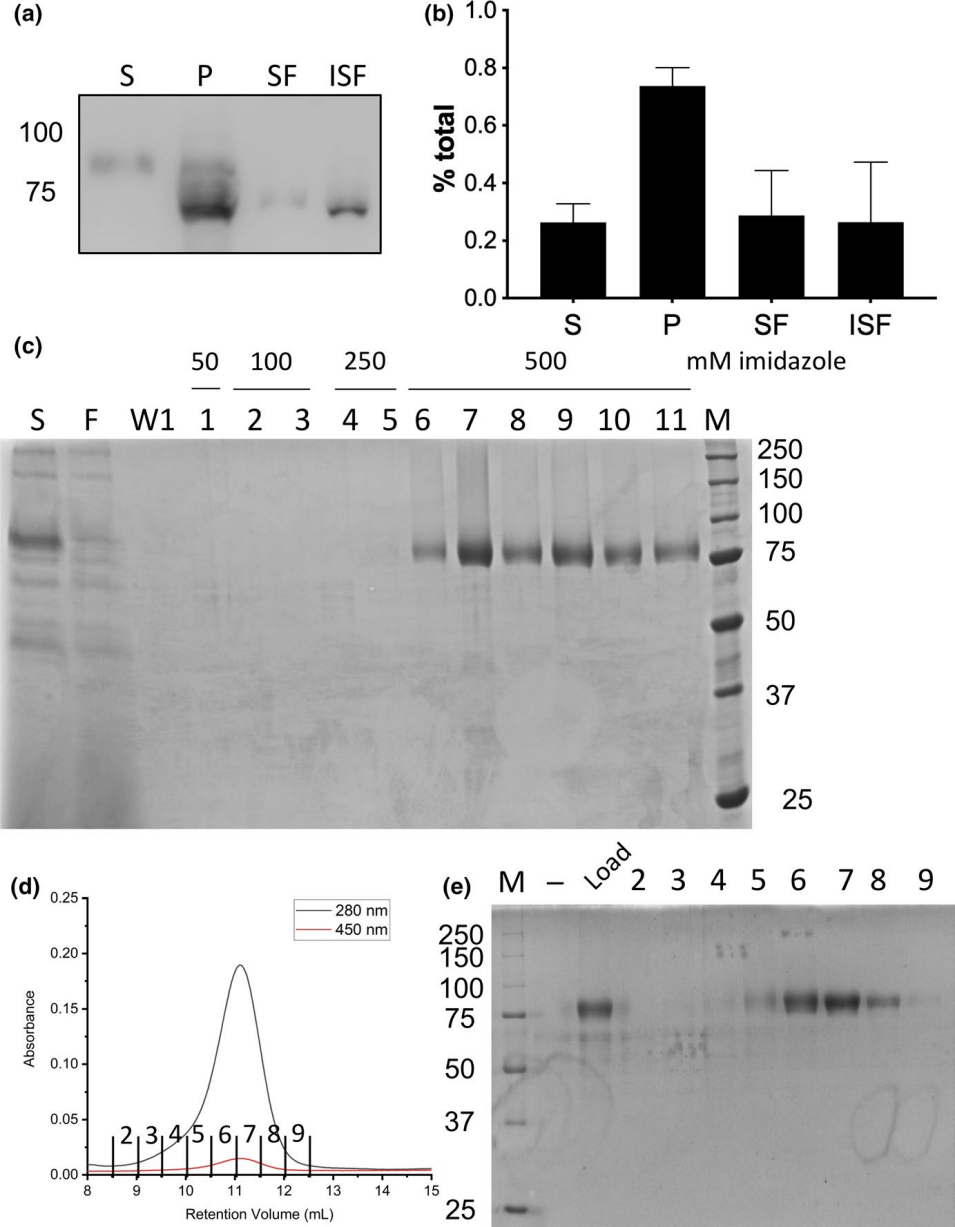
Purification of recombinant 9‐*hc*LAAO4. The construct for the 9‐*hc*LAAO4 with prepro‐⍺‐factor ER signal sequence was integrated into the genome of *P*.* pastoris* SMD1163. 9‐*hc*LAAO4 fusion protein was expressed at 15°C, pH 6 and 1% methanol (final concentration (v/v), added every 24 hr) for 72 hr. (a) The expression was analyzed by Western blotting. A secreted enzyme could be found in supernatant (S). The pellet (P) contained the cells including cytosolic proteins. The pellet was homogenized and separated into a soluble fraction (SF) and an insoluble fraction (ISF). (b) The fractions were quantified using Image J (*n* = 6; error bars represent standard deviations). (c) The culture medium (S) was loaded onto Ni^2+^‐NTA resin. After collection of the flow‐through (F) and wash fractions (W1), proteins were eluted using increasing concentrations of imidazole (1–11). Fractions were separated by SDS‐PAGE, and proteins were stained with Coomassie. (d) Untreated 9‐*hc*LAAO4 was separated by size exclusion chromatography. It eluted at a retention volume of 11.5 ml corresponding to a molecular mass of 216 kDa. Protein was measured as absorbance at 280 nm (black) and FAD as absorbance at 450 nm (red). (e) Fractions were analyzed by SDS‐PAGE. The column load was diluted 1:10

Recently, we described the successful expression of 6‐*hc*LAAO4 in *E*.* coli* Arctic Express (DE3; Bloess et al., 2019). Now, we describe the expression of *hc*LAAO4 with an N‐terminal 9‐tag in *Pichia pastoris*. The synthetic gene encoding LAAO4 from the *Basidiomycota Hebeloma cylindrosporum* lacks the information for the putative signal sequence (GenBank accession number MH751433) and was codon‐optimized for expression in *P*.* pastoris*. The gene was cloned into the *P*.* pastoris* expression vector pPIC9K which encodes the prepro‐sequence of the α‐mating factor of *S*.* cerevisiae* as an N‐terminal ER import signal sequence. pSBL11 was integrated into the *HIS4* locus of *P*.* pastoris* SMD1163 cells. Clones were selected using G‐418 as the resistance against G‐418 is proportional to the integrated expression cassettes (Scorer et al., 1994). The optimal parameters for expression of 9‐*hc*LAAO4 were identified as a temperature of 15°C, a pH‐value of 6.0, and a methanol concentration of 1% for 72 hr. About 25% of 9‐*hc*LAAO4 was secreted into the cell medium and could be found in the supernatant (Figure 1a,b, fraction S) whereas about 75% were found in the cell pellet fraction (Figure 1a,b, fraction P). After the rupture of the cells, low‐speed centrifugation was used to separate soluble proteins and small organelles (fraction SF) from larger particles (fraction ISF). 9‐*hc*LAAO4 was detected in similar amounts in both fractions. The intracellular enzyme had higher mobility compared to the secreted form. These data indicate that 9‐*hc*LAAO4 obtained posttranslational modifications before secretion. Ni^2+^‐NTA resin was used to purify the enzyme from the culture medium, the supernatant obtained after removing the cells by centrifugation (Figure 1c, fraction S). Only low amounts of 9‐*hc*LAAO4 did not bind to the resin and could therefore not be purified (Figure 1c, fraction F). In the washing fraction, as well as the first elution fractions, no protein could be eluted (Figure 1c, fractions W1, 1–5). 9‐*hc*LAAO4 of high purity was obtained with 500 mM imidazole (Figure 1c, fractions 6–11). The purified enzyme was subjected to size exclusion chromatography (Figure 1d,e). It eluted as a single peak corresponding to a molecular mass of 216 kDa. The FAD loading of the enzyme expressed in *P*.* pastoris* was 80%–100% in different expressions (Figure A4), as determined spectroscopically (see (Hahn, Neumeister, et al., 2017)) indicating that the enzyme is folded properly for cofactor binding. In total, we obtained 4 mg of the enzyme with a total activity of about 90 U for the untreated enzyme from a 500 ml culture.

### 3.2 Activity and pH optimum of 9‐*hc*LAAO4

**FIGURE 2:**
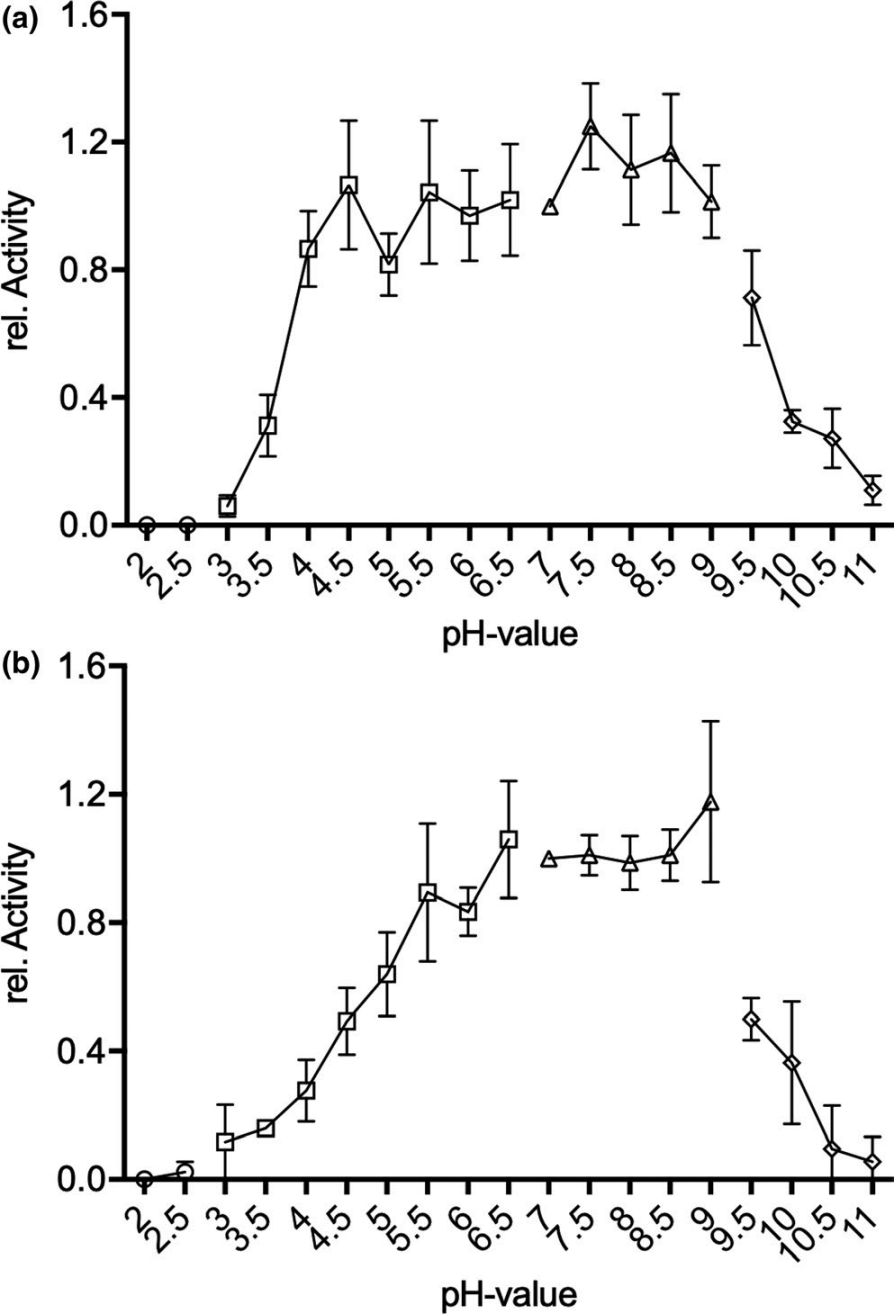
pH optimum of 9‐*hc*LAAO4. Enzymatic activity was assayed with peroxidase and *o*‐dianisidine in a glycine‐HCl buffer (circles), citric acid buffer (squares), TEA buffer (triangles), and glycine buffer (diamonds) at the indicated pH with l‐glutamine as substrate. (a) The untreated 9‐*hc*LAAO4 showed a broad maximum between pH 4 and 9 (10–20 U/mg). (b) 9‐*hc*LAAO4 was preincubated for 10 min at pH 3 before dilution into the indicated pH. The pH optimum was between pH 6 and 9 for the acid‐activated enzyme with a twofold increase in the specific activity. Data are means of three independent experiments; error bars represent standard deviations

The activity of 9‐*hc*LAAO4 was assayed using 10 mM l‐glutamine as a substrate. The initial production rate of H_2_O_2_ was measured using a coupled peroxidase/*o*‐dianisidine assay. To investigate the pH optimum of the 9‐*hc*LAAO4, the purified enzyme was incubated with buffers at pH 2–11 (Figure 2). For the untreated enzyme, the activity remains on the same level in the range of pH 4–9 (Figure 2a). The specific activities of 9‐*hc*LAAO4 in untreated conditions varied between 10 and 20 U/mg while only 2 U/mg were observed for 6‐*hc*LAAO4 expressed in *E*.* coli* (Bloess et al., 2019). As the *E*.* coli* expressed enzyme was activated by incubation for 10 min at pH 3.0, this procedure was also tested for 9‐*hc*LAAO4 expressed in *P*.* pastoris*. With acid treatment, the specific activity could be increased twofold up to 20–40 U/mg. This specific activity is similar to the one obtained for the activated *E*.* coli* expressed enzyme. A pH optimum between pH 6–9 could be detected for the acid‐activated (pH 3) enzyme (Figure 2b). A complete loss of activity for a pH under 3 could be determined for both conformations. With more basic pH‐values, a decrease in activity was observed.

### 3.3 Glycosylation of 9‐*hc*LAAO4

**FIGURE 3:**
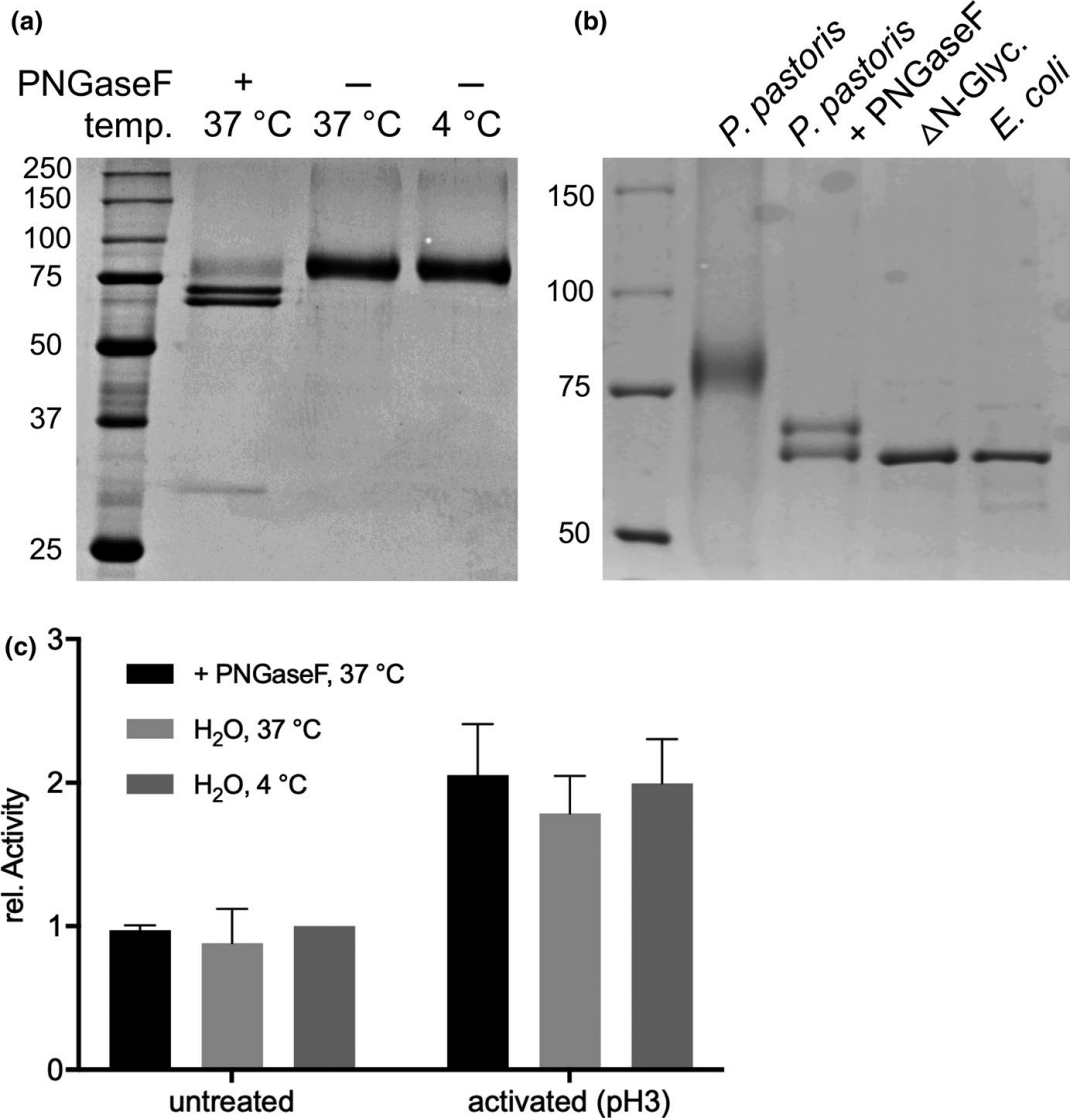
Glycosylation of 9‐*hc*LAAO4 expressed in *P*.* pastoris*. (a) Purified 9‐*hc*LAAO4 was treated with PNGaseF overnight at 37°C to remove the glycan structure from the fusion protein or incubated at 37°C without additions. To exclude that the temperature had an impact, the enzyme was stored at 4°C. The separation was performed by SDS‐PAGE, and proteins were stained with Coomassie. (b) Untreated 9‐*hc*LAAO4 (*P*.* pastoris*), deglycosylated 9‐*hc*LAAO4 (*P*.* pastoris* +PNGaseF), 9‐*hc*LAAO4 without any predicted glycosylation sites (△N‐Glyc.), and untreated 6‐*hc*LAAO4 (*E*.* coli*) were separated by Coomassie‐stained SDS‐PAGE. (c) Deglycosylation did not reduce the activity of the 9‐*hc*LAAO4. 9‐*hc*LAAO4 was incubated as indicated above. Enzymatic activity was assayed with peroxidase and *o*‐dianisidine in TEA buffer pH 7 for untreated 9‐*hc*LAAO4 and acid‐activated fusion protein with l‐glutamine as substrate. Data are means of three independent experiments; error bars represent standard deviations. The data were normalized to the 4°C control of the untreated enzyme

**FIGURE A1:**
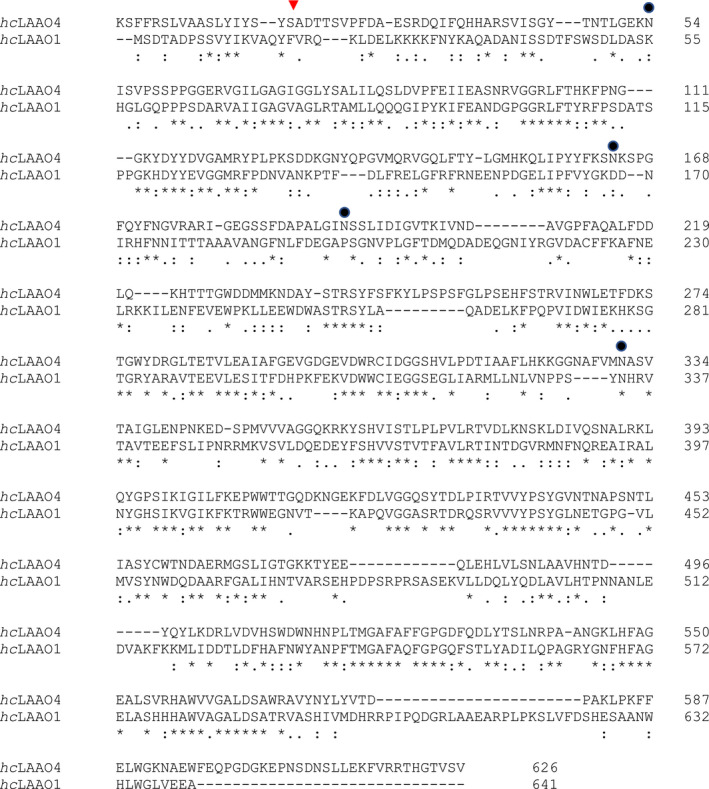
Sequence alignment of *hc*LAAO4 with *hc*LAAO1. Alignment was done via Clustal Omega web tool. Predicted N‐glycosylation sites (NetNGlyc 1.0 Server) for *hc*LAAO4 are marked (N54, N164, N193, N331, black circles). The predicted cleavage site of the N‐terminal signal sequence of *hc*LAAO4 is shown (red arrow) and marks the start of the sequence included in 9‐ *hc*LAAO4

**FIGURE A2:**
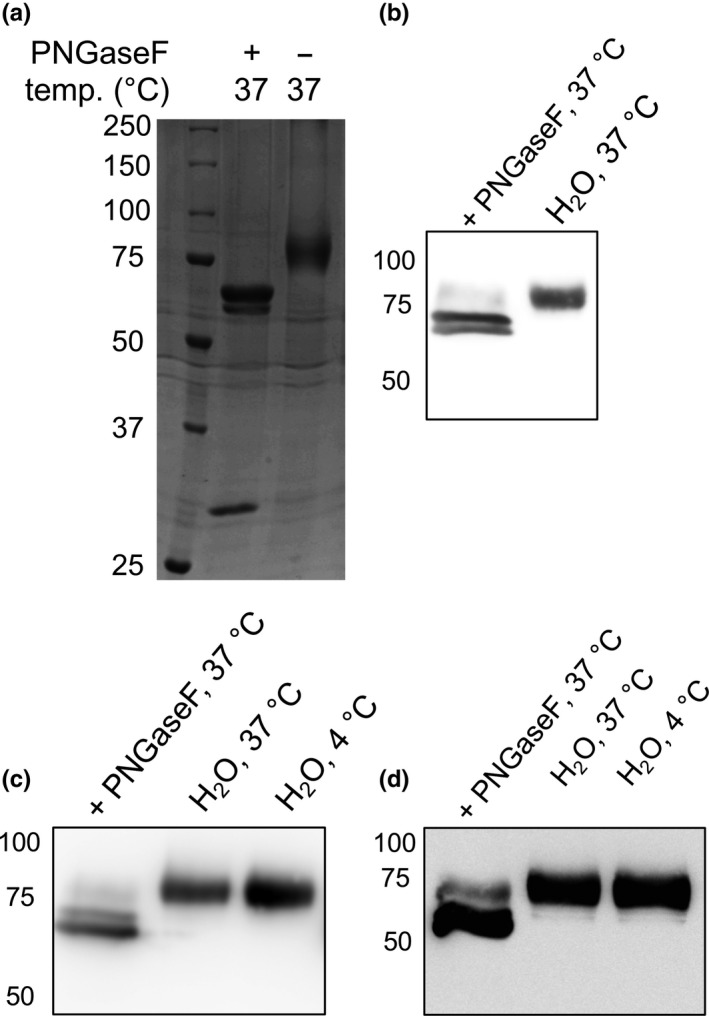
Deglycosylation of different batches of 9‐*hc*LAAO4. Deglycosylation was performed due to treatment of purified *hc*LAAO4 with PNGaseF over night at 37°C. The lower band appearing after deglycosylation (+ PNGaseF, 37°C) represents a fully deglycosylated enzyme, whereas the upper band is partially degylcosylated. The ration between both bands varied in different experiments suggestive of variable deglycosylation efficiencies. Separation was done by SDS‐PAGE and detection by Coomassie brilliant blue staining (a) or Western blotting (b–d)

Expressed in *E*.* coli*, the 6‐*hc*LAAO4 fusion protein has a mobility of about 65 kDa in SDS‐gels (Bloess et al., 2019). This is in agreement with the estimated molecular weight of about 69.6 kDa (determined via ExPASy ProtParam). Due to an apparent molecular weight slightly above 75 kDa, 9‐*hc*LAAO4 may be glycosylated when expressed in *P*.* pastoris*. It is known that snake venom LAAOs and a secreted LAAO from the fungus *Trichoderma harzianum* are glycosylated (Pollegioni et al., 2013; Yang et al., 2011). The sequence of *hc*LAAO4 contains four putative N‐glycosylation sites predicted by the NetNGlyc program at amino acid residues N54, N164, N193, and N331 (Figure A1). Deglycosylation of purified 9‐*hc*LAAO4 was performed by adding PNGaseF. After incubation, two lower molecular weight bands could be obtained compared to the control (Figure 3a, left and middle lane). Compared to 6‐*hc*LAAO4 expressed in *E*.* coli* (Figure 3b, right lane), the lower band of the deglycosylated enzyme is on the same height, whereas a second band is slightly higher (Figure 3b, middle lane). To analyze this further, all four asparagine residues with the potential to become N‐glycosylated were changed to alanine residues and the construct expressed in *P*.* pastoris*. This purified enzyme has the same mobility as the lower band of 9‐*hc*LAAO4 treated with PNGaseF (Figure 3b lane ∆N‐Glyc). These data indicate that the lower band represents a fully deglycosylated 9‐*hc*LAAO4 while the upper band obtained in different amounts in different PNGaseF treatments (Figure A2) is partially deglycosylated. Furthermore, without activation, higher specific activity was obtained when the enzyme was expressed in *P*.* pastoris* compared to *E*.* coli*. Therefore, we wondered whether glycosylation increases enzymatic activity. The activity was determined after treatment with and without PNGaseF for the untreated and the acid‐activated enzyme (Figure 3c). Neither the untreated enzyme nor the activated (pH 3) enzyme showed a loss of activity after deglycosylation.

### 3.4 Enzymatic properties of 9‐*hc*LAAO4

Next, we wanted to test, whether the expression system affected the substrate scope. The best substrates for untreated 9‐*hc*LAAO4 were l‐glutamine followed by l‐leucine, other hydrophobic l‐amino acids, l‐glutamic acid, and basic l‐amino acids at a substrate concentration of 10 mM (Table 1). Amino acids with a branched β‐position were hardly accepted as substrates (l‐valine) or not converted (l‐*tert*‐leucine and l‐phenlyglycine). Glycine and all tested d‐amino acids were not accepted. Interestingly, amino acids like dl‐homophenylalanine and 4‐chloro‐dl‐phenylalanine were converted with 44 and 34% relative activity compared to l‐glutamine (Table 1). Even methyl and ethyl esters were substrates for the 9‐*hc*LAAO4 expressed in *P*.* pastoris*. There was no big difference in substrate scope for untreated and acid‐activated 9‐*hc*LAAO4.

**TABLE 1:** Substrate scope of 9‐*hc*LAAO4

	Substrates	Relative activity (%)
Hydrophobic amino acids	l‐alanine	28
	l‐isoleucine	20
	l‐leucine	74
	l‐*tert*‐leucine	0
	l‐norleucine	28
	l‐methionine	45
	l‐phenylalanine	42
	l‐phenylglycine	0
	rac‐β‐phenylalanine	0
	l‐tryptophane	0
	l‐proline	8
	l‐valine	5
Polar amino acids	l‐asparagine	20
	l‐cysteine	0
	l‐glutamine	100
	l‐serine	0
	l‐threonine	9
	l‐tyrosine^a^	25
Basic amino acids	l‐arginine	20
	l‐histidine	29
	l‐lysine	21
	l‐ornithine	41
Acidic amino acids	l‐aspartic acid	1
	l‐glutamic acid	29
Amino acid derivates	l‐alanine ethyl ester	5
	l‐glutamic acid dimethyl ester	32
	l‐leucine methyl ester	54
	l‐leucine ethyl ester	49
	l‐methionine methyl ester	40
	l‐phenylalanine methyl ester	27
	l‐tyrosine methyl ester	7
	l‐threonine methyl ester	0
	l‐ß‐alanine ethyl ester	0
	ß‐alanine	0
	l‐DOPA^a^	8
	dl‐homophenylalanine^a^	44
	4‐chloro‐dl‐phenylalanine	34

Note

Standard substrate concentration: 10 mM.

^a^ 2.5 mM was used because of low solubility.

Kinetic properties were determined for untreated and acid‐activated 9‐*hc*LAAO4 by measuring initial velocities at different substrate concentrations and calculations from Hanes–Woolf plots (Table 2). l‐glutamine, l‐leucine, l‐phenylalanine, and l‐methionine were tested as well as the methyl esters of l‐leucine and l‐phenylalanine. *K*_m_‐values were below 1 mM for these free amino acids and barely changed after acid activation. These results indicated that activation did not change the substrate affinity of the enzyme. Only the two tested methyl esters showed a lower affinity (*K*_m_‐value 1.85 mM for l‐phenylalanine methyl ester and 3.0 mM for l‐leucine methyl ester) for the untreated enzyme. The *v*_max_ values increased about threefold due to acid activation.

**TABLE 2:** Kinetic properties of untreated (left) and acid‐activated (right) 9‐*hc*LAAO4. *K*_m_ and *v*_max_ were calculated from Hanes–Woolf plots. Data are means of three independent experiments with standard deviations

	Untreated		Activated (pH 3)	
	*K*_m_ (mM)	*v*_max_ (U/mg)	*K*_m_ (mM)	*v*_max_ (U/mg)
l‐glutamine	0.51 ± 0.11	8.87 ± 0.51	0.59 ± 0.05	27.86 ± 6.01
l‐leucine	0.04 ± 0.04	7.67 ± 0.50	0.29 ± 0.03	28.21 ± 0.81
l‐phenylalanine	0.07 ± 0.07	3.25 ± 0.11	0.07 ± 0.04	12.11 ± 0.80
l‐phenylalanine methyl ester	1.85 ± 0.60	2.86 ± 0.13	1.12 ± 0.36	9.21 ± 1.79
l‐leucine methyl ester	3.01 ± 0.68	8.41 ± 1.21	3.58 ± 2.44	22.32 ± 4.91
l‐methionine	0.16 ± 0.10	5.98 ± 0.38	0.41 ± 0.17	14.31 ± 2.37

**FIGURE 4:**
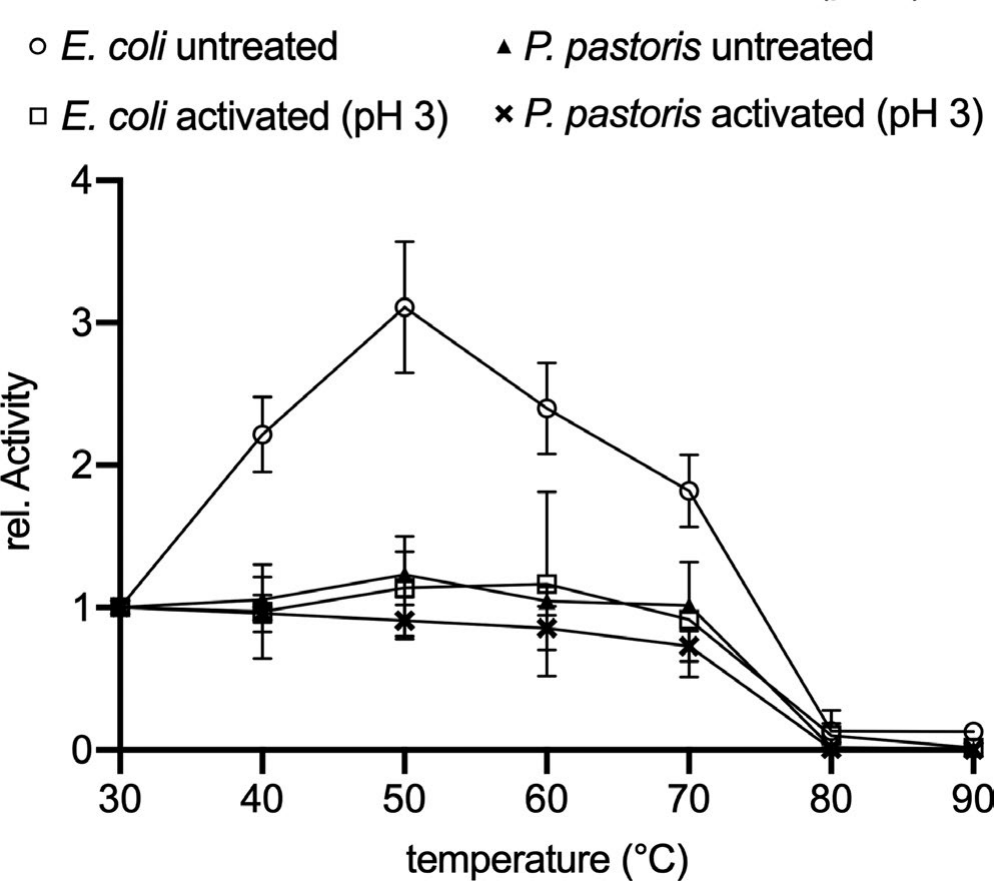
Temperature stability of 6‐*hc*LAAO4 expressed in *E*.* coli* (open circles (untreated) and open squares (acid‐activated)) and 9‐*hc*LAAO4 expressed in *P*.* pastoris* (filled triangles (untreated) and crosses (acid‐activated)). Enzymes were incubated for 1 hr at the indicated temperature and assayed immediately with peroxidase and *o*‐dianisidine in TEA buffer pH 7 at 30°C with l‐glutamine as substrate. Enzyme expressed in *E*.* coli* or *P*.* pastoris* was stable up to 70°C. Untreated enzyme expressed in *E*.* coli* was activated by incubation between 40°C and 70°C. Data are means of three (*E*.* coli* enzymes) or six (*P*.* pastoris* enzymes) independent experiments; error bars represent standard deviations

To determine the effect of the expression system and acid activation on the stability of *hc*LAAO4, we incubated the enzymes for an hour at various temperatures. Activities were assayed by coupled peroxidase/*o*‐dianisidine assay (Figure 4). 9‐*hc*LAAO4 expressed in *P*.* pastoris* was stable up to 70°C independent of acid treatment. Their stability was similar to acid‐activated 6‐*hc*LAAO4 expressed in *E*.* coli*. Interestingly, untreated 6‐*hc*LAAO4 showed an increase in activity with a maximum at 50°C compared to 30°C. However, the specific activity was sixfold lower at 50°C compared to the acid‐activated enzyme. At 80°C, a total loss of activity for all enzymes could be detected.

### 3.5 High cell density fermentation of *E*.* coli* and *P*.* pastoris* in bioreactors

**FIGURE 5:**
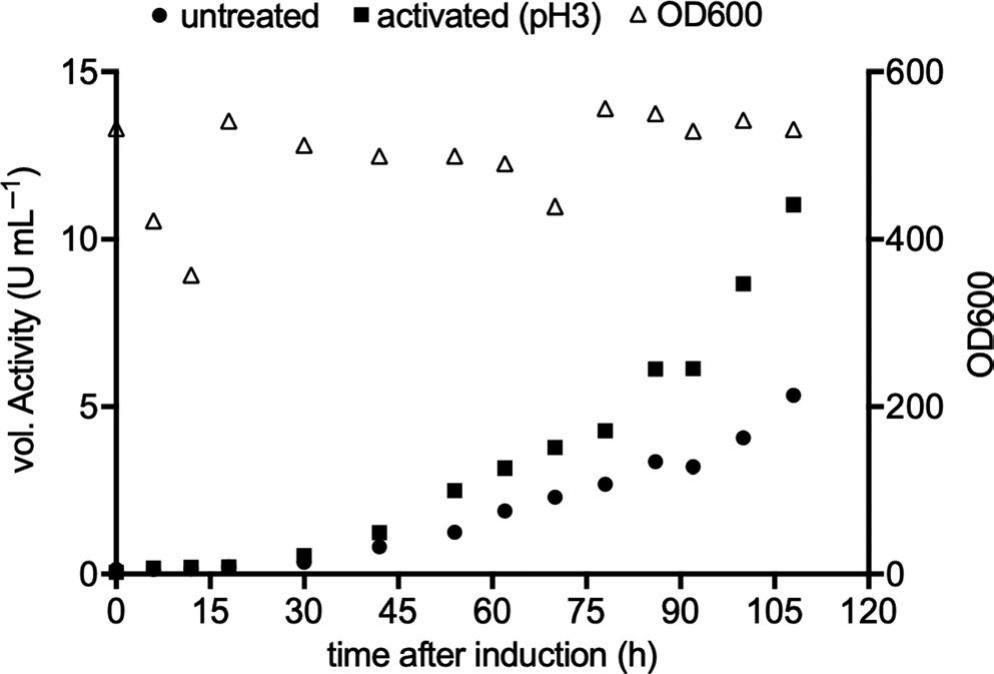
Temporal progression of cell density (OD600) and activity of expressed 9‐*hc*LAAO4 in a bioreactor after induction. Expression was carried out at 15°C and pH 6 for 108 hr in a bioreactor. Final methanol concentration was regulated with the pO_2_ value. Cell density was measured by determining the absorbance of the culture (open triangles). Samples were taken and centrifuged, and enzymatic activities were assayed in the untreated supernatants (circles) and pH 3 treated supernatants (squares) with peroxidase, *o*‐dianisidine, and l‐glutamine at 30°C

To obtain higher amounts of recombinant 9‐*hc*LAAO4, we grew *P*.* pastoris* to high cell density in a fermenter culture. *P*.* pastoris* is known for its high cell density fermentation to produce recombinant proteins (Siegel & Brierley, 1989). *P*.* pastoris* was grown to a cell density of about 500 OD600 (up to 112 g/L dry cell weight concentration) before induction of 9‐*hc*LAAO4 expression by methanol for 4–5 days in a bioreactor. The time course of 9‐*hc*LAAO4 activity as well as the cell density was followed during *P*.* pastoris* bioreactor cultivation (Figure 5). Whereas the cell density remained the same during fermentation (about 450–550), the activity in the supernatant increased steadily. The activity in the supernatant reached 9200 U/L compared to 180 U/L in shaking flask cultures (Table 3).

**TABLE 3:** Comparison of yield and activity of *hc*LAAO4 expressed in a shaking flask or a bioreactor for both expression systems

	*E. coli*		*P. pastoris*	
	Shaking flask	Bioreactor	Shaking flask	Bioreactor
volume	0.5 L	1.7–2.3 L	0.5 L	2.4 L
OD600	10	38–92	50	500
Activity per dry mass	455 U/g	175–260 U/g	9 U/g	44 U/g
Activity per liter	2000 U/L	2800–10,000 U/L	180 U/L	9200 U/L

We also fermented *E*.* coli* Arctic Express (DE3) to increase the yield of recombinant 6‐*hc*LAAO4. Various cultivation conditions for cell growth before induction were tested, whereby the best result was performed after a 47 hr growth phase in batch procedures at 30°C and an induction time of 25 hr. While cell densities were higher after fermentation compared to shaking flask cultures, the activity per gram dry mass was lower (Table 3). Compared to *P*.* pastoris*, induction periods for the expression in *E*.* coli* were significantly shorter (110 hr vs. 25 hr).

## 4 DISCUSSION

**FIGURE A3:**
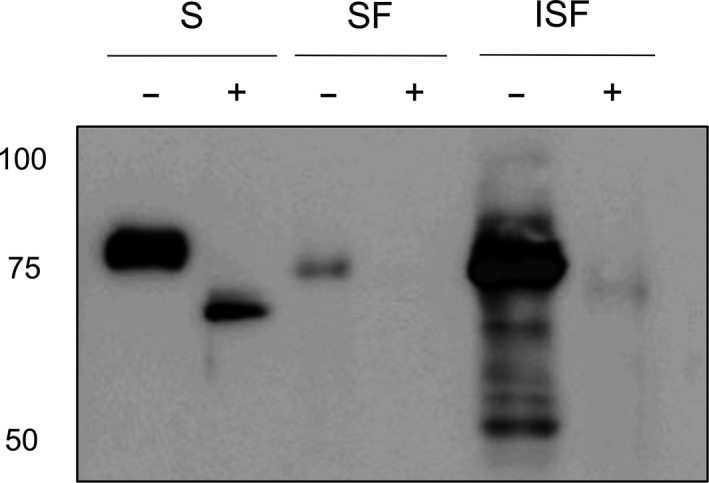
Deglycosylation of different fractions of 9‐*hc*LAAO4. Secreted proteins (S) as well as lysed cell pellets separated into a soluble fraction (SF) and an insoluble fraction (ISF) were used. Deglycosylation was performed by treatment of *hc*LAAO4 with PNGaseF over night at 37°C. Fractions were separated by SDS‐PAGE and 9‐*hc*LAAO4 detected by Western blotting. The enzyme within the cells (band in lane ISF, −) is resistant to PNGaseF (weak band in lane ISF, +) indicating that it is not glycosylated. −: without PNGaseF +: with PNGaseF

**FIGURE A4:**
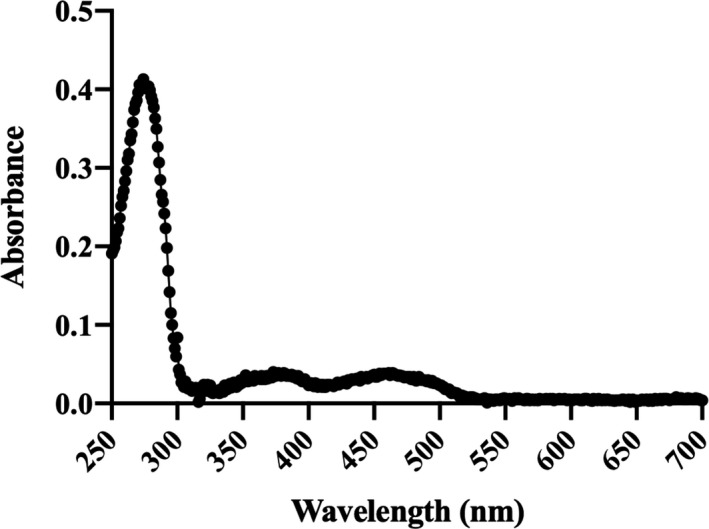
UV‐Vis Spectrum of untreated 9‐*hc*LAAO4. The absorption spectrum between 350 and 500 nm is typical for flavoproteins. Cofactor occupancy was calculated from the ratio of the absorbance at 280 and 450 nm. The loading was 80%–100% in different expressions. Spectra were determined from three different experiments as described (Hahn, Neumeister, et al., 2017)

Even though *H*.* cylindrosporum* is a fungus (*Basidiomycota*), we were able to express *hc*LAAO4 without its ER signal sequence in the cytosol of *E*.* coli* (Bloess et al., 2019). The yeast *Pichia pastoris* (*Ascomycota*) has some major advantages over bacterial expression systems such as posttranslational modifications in ER and Golgi followed by secretion into the medium (Cereghino & Cregg, 2000). l‐amino acid oxidases from snake venom and flounders have already been expressed in *P*.* pastoris* as secreted proteins (Kasai et al., 2015; Kommoju, Macheroux, & Ghisla, 2007). Here, we describe that by adding the prepro‐sequence of the α‐factor from *S*.* cerevisiae*, recombinantly expressed 9‐*hc*LAAO4 was secreted into *P*.* pastoris* culture medium. A faster and more economical purification of the enzyme could be achieved with this expression system. However, 75% of 9‐*hc*LAAO4 was detected in the cell pellet at an apparent molecular mass of about 75 kDa in SDS‐PAGE in shaking flask cultures (Figure 1). The apparent molecular mass of the intracellular form was lower than that of the secreted enzyme suggesting the addition of posttranslational modifications within the secretory pathway for the secreted form. Glycosylation of the secreted enzyme was demonstrated by the reduction of the apparent molecular mass to about 70 kDa after incubation with an N‐deglycosylating enzyme (Figure 2). The position of the lower band was at the same height as the enzyme expressed in *E*.* coli* indicating that it did not contain further modifications, which affect mobility. This was confirmed by the expression of a secreted 9‐*hc*LAAO4 variant without predicted N‐glycosylation sites, which had the same mobility on the SDS gel. The enzyme within the cells is resistant to N‐glycosidase F (Figure A3) indicating that it is not glycosylated. The apparent molecular mass of about 75 kDa for the form within the cells compared to 70 kDa for the unglycosylated, secreted enzyme suggests that it still contains the prepro‐α‐factor sequence of a calculated molecular mass of 9.9 kDa, which is removed in the Golgi apparatus by a protease. Therefore, the enzyme remaining within the cells should reside in the cytosol due to misfolding or limitations in the ER import machinery.

Glycosylation was described for snake venom LAAOs (Geyer et al., 2001; Hayes & Wellner, 1969) and a secreted LAAO from the fungus *Trichoderma harzianum* ETS 323 belonging to *Ascomycota* (Yang et al., 2011). Due to this glycosylation of a secreted fungal LAAO, the glycosylation of 9‐*hc*LAAO4 in the yeast *P*.* pastoris* and its native signal sequence for ER import we assume that this enzyme is also glycosylated in *H*.* cylindrosporum*.

Enzymes expressed in *P*.* pastoris* and *E*.* coli* could both be activated by brief exposure to acidic pH and reached similar specific activities after acid activation. The kinetic parameters *K*_m_ and *v*_max_ were also similar. However, the expression of the enzyme in *P*.* pastoris* resulted in a higher specific activity without activation. 9‐*hc*LAAO4 expressed in *P*.* pastoris* showed *v*_max_ values of 9 U/mg for l‐glutamine, whereas the activity of the enzyme expressed in *E*.* coli* displayed 2 U/mg for this substrate (Bloess et al., 2019). Deglycosylation of untreated 9‐*hc*LAAO4 expressed in *P*.* pastoris* did not reduce activity. These data argue against a direct influence of N‐glycosylation on activity. However, glycosylation may induce a conformation of intermediate activity during ER import, which is stable even after the removal of the N‐glycans. Alternatively, there may be an additional posttranslational modification, which is not evident by the mobility shift in SDS‐PAGE.

The enzyme is stable during a preincubation for one hour at a temperature of up to 70°C independent of expression in *E*.* coli* or *P*.* pastoris* or treatment with acidic pH. Untreated 6‐*hc*LAAO4 expressed in *E*.* coli* was even more active after preincubation at temperatures between 40°C and 70°C compared to a preincubation at 30°C. These data indicate that this enzyme can be transformed into a more active conformation by heat in addition to freezing, acid treatment, and SDS addition as reported before (Bloess et al., 2019). Heat activation has also been observed before. Tyrosine aminotransferase from chick liver can undergo repeated cycles of reversible heat activation and cold inactivation (Shioji et al., 1978). Lysozyme form Hen's eggs can also be activated reversibly by heat (Hayashi, Funatsu, & Hamaguchi, 1963).

The substrate scope of 9‐*hc*LAAO4 expressed in *P*.* pastoris* is nearly the same as for 6‐*hc*LAAO4 expressed in *E*.* coli* (Bloess et al., 2019) indicating that glycosylation is without influence. *hc*LAAO4 has a broader substrate scope independent of expression in *E*.* coli* or *P*.* pastoris* compared to *hc*LAAO1 (Nuutinen, Marttinen, Soliymani, Hilden, & Timonen, 2012) and MBP‐*rs*LAAO1 (Hahn, Neumeister, et al., 2017).

In comparison with shaking flask cultures (4 g/L), fermentation of *E*.* coli* in bioreactors leads to a higher cell dry mass per L (16–39 g/L) with a higher activity per L (2800–10,000 U/L compared to 2000 U/L). In contrast, the activity per g dry mass was lower (175–260 U/g and 455 U/g). However, results in fermentation were variable regarding enzyme yields and activities. Various cultivation conditions for the growth of the *E*.* coli* cells were tested, whereby batch procedures at 30°C gave the best results. This is likely to be due to the better plasmid stability under these conditions. But the results in fermentation were variable regarding enzyme yields and activities even with the same cultivation conditions. Also, realizing higher dry cell mass concentrations by high cell density fermentation was limited due to decreasing plasmid stability over time. Moreover, biomass yields regarding the medium‐dry mass are rather low (0.14 for fed‐batch fermentation), indicating cell stress, probably caused by the depletion of l‐amino acids and the production of toxic H_2_O_2_ by *hc*LAAO4. Because of shorter cultivation times, the productivity of *hc*LAAO4 was higher for *E*.* coli* compared to *P*.* pastoris*.

Fermentation of 9‐*hc*LAAO4 in *P*.* pastoris* yielded a 50‐fold higher activity per liter (9200 U/L) in the medium compared to expression in a shaking flask (180 U/L). One reason is the 10‐fold higher cell density, which can be obtained by fermentation in a bioreactor. The others are the controlled feeding and the prolonged cultivation time of 4.5 days possible in fermentation compared to 3 days in the shaking flasks. Besides, *hc*LAAO4 was secreted into the extracellular environment protecting the cells from the depletion of l‐amino acids and the production of toxic H_2_O_2_ as well as facilitating down‐stream processing. Thus, *P*.* pastoris* is an excellent expression system to produce recombinant *hc*LAAO4 in large quantities for industrial use.

## CONFLICT OF INTEREST

None declared.

## AUTHOR CONTRIBUTIONS

**Marc Christian Heß:** Conceptualization (supporting); Data curation (supporting); Investigation (lead); Writing‐original draft (lead). **Svenja Bloess:** Conceptualization (supporting); Data curation (supporting); Investigation (supporting); Writing‐review & editing (supporting). **Joe Max Risse:** Conceptualization (supporting); Data curation (supporting); Investigation (supporting); Writing‐review & editing (supporting). **Karl Friehs:** Conceptualization (supporting); Data curation (supporting); Resources (equal); Supervision (supporting); Writing‐review & editing (supporting). **Gabriele Fischer von Mollard:** Conceptualization (lead); Data curation (supporting); Resources (equal); Supervision (lead); Writing‐review & editing (lead).

## ETHICS STATEMENT

Work with recombinant DNA has been performed according to national requirements (Dt‐55.3.5‐5/94‐Bi, Anlage Nr. 412).

## Data Availability

Data associated with this article can be found at “Publikationen an der Universität Bielefeld” (PUB): https://doi.org/10.4119/unibi/​2944932

## Appendix 1

**TABLE A1:** Overview of primers used for overlap PCR to change predicted N‐glycosylation sites to alanine residues

Primer	Sequence (5'–3’)
hc_N54A_fwd1	CTAACACTTTGGGTGAGAAGGCCATCTCTGTTCCATCTT
hc_N54A_rev1	TGGAGAAGATGGAACAGAGATGGCCTTCTCACCC
hc_N193A_fwd2	TCCAGCTTTGGGTATCGCCTCCTCCTTGATTGA
hc_N193A_rev2	GATGTCAATCAAGGAGGAGGCGATACCCAAAGCT
hc_N331A_fwd3	GGTAACGCTTTCGTTATGGCCGCTTCCGTTACT
hc_N331A_rev3	ATAGCAGTAACGGAAGCGGCCATAACGAAAGCGTTA
hc_N164A_for4	ACTACTTCAAGTCCGCCAAGTCCCCAGGTTTC
hc_N164A_rev4	CTGGAAACCTGGGGACTTGGCGGACTTGAAGT
EcoRI_for1	AAGAATTCTTCGAAGGATCCAAACGATGAGAT
Not1‐HH_r2	AAGGCGGCCGCTTAAACGGAAAC
